# Monomer exchange dynamics in ureido–pyrimidinone supramolecular polymers *via* molecular simulations

**DOI:** 10.1039/d5tb01272d

**Published:** 2025-09-24

**Authors:** Annalisa Cardellini, Cristina Caruso, Laura Rijns, Patricia Y. W. Dankers, Giovanni M. Pavan, Claudio Perego

**Affiliations:** a Department of Innovative Technologies, University of Applied Sciences and Arts of Southern Switzerland via La Santa 1 Lugano Switzerland annalisa.cardellini@supsi.ch claudio.perego@supsi.ch; b Department of Applied Science and Technology, Politecnico di Torino Corso Duca degli Abruzzi 24 Torino 10129 Italy; c Institute for Complex Molecular Systems, Eindhoven University of Technology 5600 MB Eindhoven P.O. Box 513 The Netherlands; d Department of Biomedical Engineering, Eindhoven University of Technology 5600 MB Eindhoven P.O. Box 513 The Netherlands; e Department of Chemical Engineering and Chemistry, Eindhoven University of Technology 5600 MB Eindhoven P.O. Box 513 The Netherlands

## Abstract

The use of synthetic supramolecular polymers, built with monomers that self-assemble *via* non-covalent, reversible interactions, is rapidly growing in many fields, including energy, environmental, and bioengineering applications. Very recently, ureido–pyrimidinone (UPy)-based supramolecular polymers have been used to synthesize biocompatible hydrogels aiming to mimic the dynamic environment of extracellular matrices. Tuning the dynamics, stiffness, and bioactivity of UPy hydrogels effectively influences cellular behaviour and tissue development. However, a complete understanding of UPy-network dynamics over different length and time scales is still lacking, and even the most advanced experimental approaches are unable to capture the dynamics of monomer exchange with atomistic resolution. Here we present a computational study on UPy supramolecular assemblies in water that uncovers the mechanism of monomer exchange between the UPy supramolecular polymers and their surroundings. Our results, based on atomistic molecular dynamics (MD) simulations combined with enhanced sampling and machine-learning (ML) techniques, show that the fine interplay of solute–solvent interactions is the main engine of monomer motion, which makes UPy supramolecular polymer ends more dynamic as compared to the static backbone. This computational work complements the qualitative experimental evidence on supramolecular dynamics with the mechanism of monomer exchange, revealing the most favorable environment for supramolecular polymer damage as well as the underlying principle of self-healing.

## Abstract 2

A synergistic integration of molecular dynamics, enhanced sampling, and data analysis has elucidated, at atomistic resolution, the key factors governing monomer exchange dynamics in synthetic ureido-pyrimidone supramolecular polymers.

## Graphical abstract

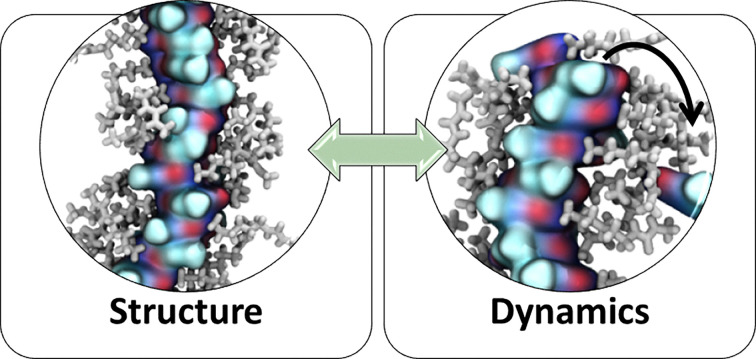

Living systems possess an intrinsic ability to mitigate various forms of damage, showing self-healing and regenerative properties.^1–3^ Inspired by such dynamic features, scientists have advanced the synthesis of functional materials with on-demand reversibility and stimuli-responsiveness.^4–8^ One common strategy relies on the design of supramolecular materials,^9^*i.e.*, assemblies of non-covalently bound monomers, holding great promise in environmental applications,^10^ drug delivery,^11–15^ regenerative medicine,^16,17^ skin-like stretchable electronics,^18,19^ and anticorrosive coatings.^20,21^ Examples of monomers forming supramolecular structures include benzene 1,3,5-tricarboxamide (BTA), self-assembled in one-dimensional (1D) supramolecular polymers *via* core–core stacking and three-fold hydrogen bonding,^22,23^ benzotrithiophene building-blocks,^24,25^ peptide amphiphiles,^26–28^ or metal-coordinated porphyrins.^29–31^ In this realm, UPy molecules have also been largely used by taking advantage of their ability to self-organize in “hierarchical” fibrillar structures in water. Indeed, UPy monomers dimerize by self-complementary quadruple hydrogen bonding in a donor–donor–acceptor–acceptor (DDAA) fashion (Fig. 1a). These planar dimers can stack on top of each other to form supramolecular polymers, (hereafter also referred to as stacks) which, in turn, can interact and bundle to form more complex fibrillar structures.^32–34^

**Fig. 1:**
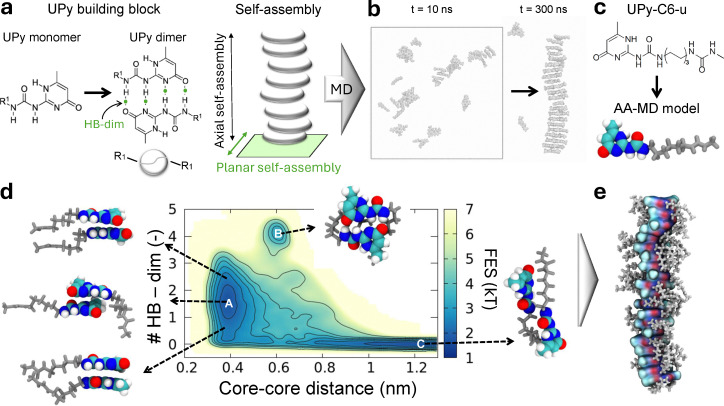
Molecular dynamics (MD) models of UPy supramolecular polymers. (a) Left: chemical structure of two UPy monomers forming a dimer by self-complementary quadruple hydrogen bonds (HB–dim). R1 indicates a possible functionalization. Right: self-assembly cartoon of UPy dimer building blocks in the axial direction, creating a supramolecular polymer. (b) All-atom MD (AA-MD) simulation of (R1-free) UPy dimer self-assembly. Two MD snapshots at 10 and 300 ns are shown. (c) Chemical structure and AA-MD model of the UPy-C6-u monomer considered in this study. Oxygen, carbon, and hydrogen atoms of the UPy-C6-u core are colored in red, gray, and white, respectively. The side chain is in light gray. (d) UPy-C6-u dimerization free-energy-surface in aqueous solution obtained with well-tempered MetaDynamics (WT-MetaD) simulation. The number of HB–dim and core–core distance are selected as the WT-MetaD collective variables. **A**, **B**, and **C** identify three energy minima corresponding to the stacking, dimerization, and lateral assembly configurations, respectively. (e) AA-MD snapshot of the pre-stacked UPy-C6-u supramolecular polymer made of 20 dimers (20D) considered in this study.

Over the past twenty years, UPy monomers have been functionalized with poly(ethylene glycol) (PEG),^35,36^ poly(*N*-isopropylacrylamide) (PNIPAM),^37,38^ glycine (Gly) amino acids,^16,39^ among others, in order to improve their biocompatibility and tunability.^40^ The resulting supramolecular polymers have demonstrated intriguing mechanical and dynamic properties in a wide range of multi-component environments, playing a crucial role in the syntheses of biomimetic hydrogels.^41,42^ For example, UPy supramolecular polymers have been exploited to mimic the liquid–liquid-phase-separation found in biological fibrils, collagen, and, in general, to reproduce the adaptive behavior of extracellular matrices with tunable stiffness, dynamics, and bioactivity.^35,39,41^ However, for inducing cell adhesion in hydrogel networks, tight control over multiscale dynamic processes is required.^43^ Particularly, a tailored understanding is crucial for both molecular-level dynamics—such as monomer exchange within a single stack—and the bulk dynamics, *i.e.*, supramolecular polymer rearrangements in hydrogels covering larger scales.

Although recent experimental studies have provided estimates for the monomer exchange rate in UPy supramolecular polymers (10% in 1 hour),^35,43^ most experimental approaches cannot resolve the molecular and submolecular mechanisms occurring in UPy self-assemblies, where monomeric and oligomeric units continuously exchange, setting a supramolecular “equilibrium dynamics.” This monomer exchange dynamics, on the other hand, can be well detected by multiscale molecular modeling and advanced computational methods.^44–46^ In this framework, while BTA supramolecular polymers have been largely investigated with atomistic,^47–49^ higher-scale simulations,^44,50,51^ and advanced ML tools,^52–54^ UPy supramolecular polymers have received much less attention from computational research. Chen *et al.*^55^ used an umbrella sampling technique to estimate the potential of mean forces between two interacting UPy molecules. While their work successfully highlights the role of the hydrophobic spacer in the UPy dimerization process, it does not capture the range of configurations that UPy building blocks may adopt while self-assembling. Later studies utilized atomistic and coarse-grained simulations to investigate the self-assembly mechanisms occurring in longer UPy supramolecular polymers.^56,57^ These approaches, however, are constrained by time and space limitations typical of classical MD, preventing a thorough exploration of the system's configurational space. Additionally, none of the computational studies on UPy self-assemblies conducted so far have investigated the essential phenomenon of monomers' exchange within and outside their self-stacking structure, underlying bioinspired properties such as self-healing and reconfiguration.

Here we present a computational work where MD, enhanced sampling approaches, and ML techniques are integrated to provide an overview of the structural and dynamics features of UPy supramolecular polymers in water. In this study, the UPy core is functionalized with short carbon spacers terminating with a urea moiety (UPy-C6-u in Fig. 1c). The UPy core forms dimers through quadruple hydrogen bonding, and the hierarchical growth is also driven by bifurcated hydrogen bonds of the urea group flanking the alkyl spacer.^58,59^ Starting from pre-assembled UPy dimers—based on literature data and preliminary MD indications—we first investigate the structural properties of these UPy dimer stacks, assessing their size-dependent stability. The dynamics of monomer exchange is then studied *via* infrequent well-tempered metadynamics (WT-MetaD) simulations,^60^ which accelerate the rupture of the dimerization hydrogen bonds across the supramolecular structure. Afterwards, we employ data-driven analyses^61,62^ to detect key dynamic environments, which reveal the mechanisms of monomers' exchange within the longest UPy stack. Finally, we also provide insights into the monomer exchange dynamics occurring between bundled UPy supramolecular polymers, the typical higher-level structure detected in experiments.^35,43,58,63^ This study provides solid guidelines to describe the structure and dynamics of UPy supramolecular assemblies in water, allowing us to gather useful indications on how the molecular structure of the system relates to the supramolecular dynamics and, subsequently, to the bioinspired properties of the material.^35,43,59^

## 1. Results

### 1.1. Structural analysis of UPy-C6-u supramolecular polymers

By using all-atom MD (AA-MD) simulations, we here describe the key physical and chemical mechanisms determining the structural stability and dynamic features of UPy supramolecular polymers. As outlined in the introduction, UPy cores dimerize in water, forming four complementary hydrogen bonds, and grow orthogonally *via* π–π stacking, building 1D supramolecular polymers (Fig. 1a). Such 1D dimer stacks then interact with each other, aggregating in fibers, which in turn form the bundle network that constitutes the supramolecular material. This hierarchical self-assembly process is shown here by using AA-MD simulations of R1-free (*i.e.*, without side-chain R1) UPy monomers. One single stack having such a stacked-dimer hierarchical structure is spontaneously formed after 300 ns-long MD simulation, starting from a suspension of 42 dispersed UPy monomers (Fig. 1b). This outcome confirms that the combination of hydrogen bonds and π–π interactions governs the mono-directional stacking of UPy cores. However, due to this high aggregation propensity, it is essential to enhance water solubility and prevent solution precipitation. Adding hydrophobic and hydrophilic side chains, *i.e.*, functional R1 groups, to the UPy core is a strategy to promote self-assembly in water and tune the resulting properties.

Building on experimental studies,^58,59^ here we select a relatively simple molecular design for the UPy motif, namely including one hydrophobic spacer with 6 carbon atoms (C6-spacer) and one urea terminal (the chemical structure of the UPy-C6-u monomer and the relative all-atom model are in Fig. 1c). This monomeric structure adds the complexity of an interacting side-chain without overly increasing the simulation times required to capture the monomer exchange dynamics across the supramolecular structure. Unlike the self-aggregation of R1-free UPy monomers (Fig. 1a), it is challenging to observe spontaneous and ordered assembly of UPy-C6-u within the timescales accessible through AA-MD. Therefore, our computational protocol includes the setup of pre-stacked UPy-C6-u supramolecular polymers based on the configurational guidelines emerging from the analysis of UPy dimerization free energy surface (FES). To estimate the FES we adopted well-tempered MetaDynamics,^64^ (WT-MetaD), choosing the number of dimerization hydrogen bonds, HB–dim, and the core–core distance as collective variables (see the Methods section). The resulting FES (Fig. 1d) exhibits three minima, **A**, **B**, and **C**, corresponding to the most probable configurations retained by two UPy-C6-u monomers in aqueous solution. The first minimum, **A**, represents the two-monomer-stacking arrangement in the orthogonal direction, having a core–core distance of ≈ 0.4 nm in conjunction with the formation of ≈ 2 HB–dim. The second minimum, **B**, corresponds to the dimer assembly driven by self-complementary quadruple hydrogen bonding in a DDAA pattern, *i.e.*, the number of HB–dim = 4, associated with a core–core distance of ≈ 0.6 nm, matching with earlier experimental results.^63^ Finally, the third minimum, **C**, lays down in the phase space of zero HB–dim and core–core distances around ≈ 1.2 nm. The MD representative snapshot of **C** state (Fig. 1d) reveals that the UPy-C6-u monomers are bound head-to-tail, held together by urea–core interactions. This suggests a possible arrangement of the inter-supramolecular polymer aggregation within a larger self-assembly. Based on these favorable dimer configurations and on the experimental evidence of supramolecular polymer structures,^32–34^ we arranged UPy-C6-u monomers in stacks formed by pre-assembled dimers, and we tested the stability of such structures *via* AA-MD (Fig. 1e).

**Fig. 2:**
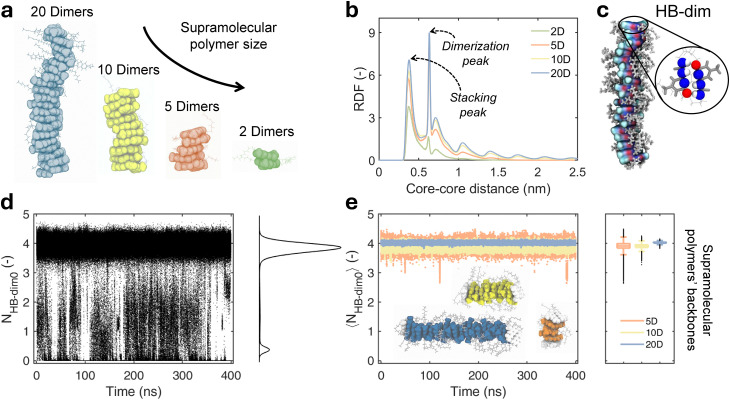
Structural analyses of UPy-C6-u stacks. (a) AA-MD snapshots of four UPy-C6-u pre-stacked supramolecular polymers of variable size: 20, 10, 5, and 2 dimers (D). (b) Radial distribution function (RDF) computed on each UPy-C6-u monomer forming the four stacks in (a). Each RDF is normalized by the number of stacked monomers. Blue, yellow, orange, and green RDFs identify the 20D, 10D, 5D, and 2D supramolecular polymers. (c) AA-MD snapshot of the 20D pre-assembled stack. The atoms chosen to account for the number of HB–dim are displayed as colored spheres in the zoomed-in view. (d) Number of initial HB–dim (*N*_HB–dim0_) time series, computed on each dimer forming the supramolecular polymers in (a) across the 400 ns equilibrated MD simulations in water. The kernel density estimation (KDE) profile of all HB–dim0 data is plotted on the right-hand side. (e) Number of HB–dim0 averaged over all the dimers in the stack backbones (tips excluded) reported in (a), as a function of *t* along the 400 ns-long MD trajectories. The 〈*N*_HB–dim0_〉 time series are colored according to the stack size; the time-averaged values of 〈*N*_HB–dim0_〉 are displayed in the boxplot on the right-hand side.

The structural stability and dynamics of the pre-assembled UPy-C6-u supramolecular polymers are investigated by simulating, for 400 ns in aqueous solution, 4 stacks of distinct sizes, *i.e.* made of 2, 5, 10 and 20 dimers (2D, 5D, 10D and 20D) (Fig. 2a). The resulting MD trajectories are firstly analyzed by computing the radial distribution function (RDF) among all monomers, considering the distance between the geometric centers of UPy cores. Regardless of stacks' length, the RDF profiles show multiple regular peaks obtained at increased core–core distance (Fig. 2b). For each system, the first peak occurs at 0.37 nm, which approximately corresponds to the position of the free energy minimum **A** (Fig. 1d). This peak therefore identifies the first neighbors along the stacking direction. The next peak, located at ≈ 0.6 nm, corresponds to the monomer–core distance relative to the dimer formation, as confirmed by the free energy landscape in Fig. 1d (**B** minimum). The subsequent lower and broader peaks include both the higher-order stacking and dimerization configurations (that are degenerate at this order). We observe that longer stacks exhibit higher peaks. Considering that the reported RDF is normalized by the number of monomers, we expect that the purely stacking peaks increase with supramolecular polymer size due to the higher statistics of neighbors. However, the same does not hold for the dimerization peak, and its size dependence is evidence of higher dimer stability in longer supramolecular polymers. We also found that the probability of a UPy-C6-u dimer to be in a perfect planar orientation is higher in a longer stack (20D) as compared to a short one (2D) (Fig. S1 in SI). These elements indicate that an ordered, planar dimer conformation is more likely in longer supramolecular polymers, suggesting cooperativity in the polymerization of UPy-C6-u.

### 1.2. Monomer exchange dynamics in UPy-C6-u supramolecular polymers

**Fig. 3:**
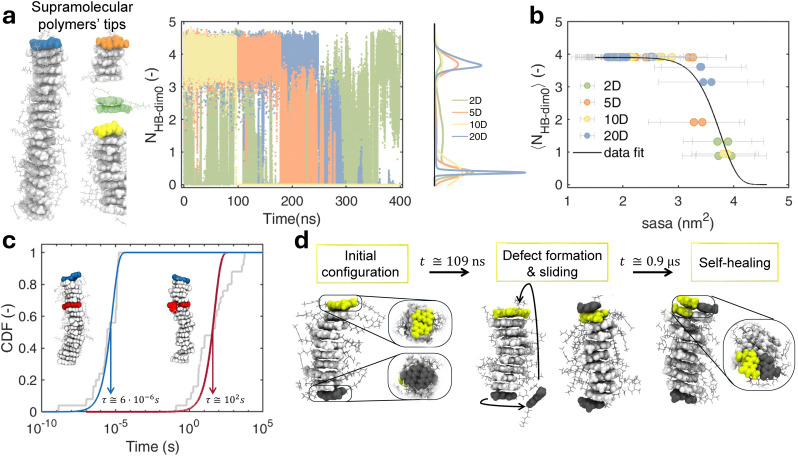
Dynamics of UPy-C6-u stacks. (a) Number of HB–dim0 time series relative to the dimers at the tips of differently sized supramolecular polymers (the considered tips are colored in the MD snapshots on the left). The KDE highlights the distribution features for the considered tip dimers, showing the breakage of the initial HBs. (b) Correlation between the time-averaged *N*_HB–dim0_ computed on each UPy-C6-u monomer forming the supramolecular polymers and its associated sasa. The data points are colored based on stack size, while a nonlinear fitted curve is shown with a black line. (c) Cumulative distribution functions (CDFs) of the defect creation times (*i.e.*, dimer rupture) resulting from 30 repetitions of infrequent WT-MetaD. Two distributions are shown, either involving a tip dimer (blue fitting curve) or a backbone dimer (red fitting curve). *τ* is the characteristic time-scale extracted *via* CDF fitting. (d) AA-MD snapshots of the 10D stack in 1 μs-long MD simulation. Initial tip dimers are highlighted in dark gray and yellow, while the supramolecular polymer backbone is in light gray. The MD snapshots show the defect formation, monomer sliding, and self-healing process involving the system.

The dynamics of supramolecular polymers is directly linked to the stability of their reversible bonds, which regulate the formation and resolution of structural defects—fundamental to self-healing mechanisms. In other words, strong non-covalent bonds among the UPy-C6-u monomers tend to prevent their reshuffling and thus the overall dynamics. In UPy-C6-u, the supramolecular polymeric structure rests both on the HB–dim forming the dimers and on the π–π stacking that fuels polymeric elongation. Therefore, to obtain quantitative insights into the dynamic nature of our UPy-C6-u pre-assembled stacks, we first focused on the reversibility of the number of HB–dim as the source of structural defects. We calculated how many of the 4 initial HB–dim (HB–dim0) are preserved by each monomer along the MD trajectories of the supramolecular polymer chains (Fig. 2a and Fig. 2c). As a result, the number of HB–dim0, *i.e. N*_HB–dim0_, of each monomer is equal to 4 at the beginning, and it decreases if the original HB–dim conformation is disrupted, as shown by the *N*_HB–dim0_ time series (Fig. 2d). As discussed in the Methods section, the estimated *N*_HB–dim0_ intrinsically includes spurious interactions that generate the fluctuations seen in the profiles (Fig. 2d). We employed relative kernel density estimation (KDE) to highlight the main features of the *N*_HB–dim0_ distribution over the MD data. In particular, the KDE evidences the presence of two main peaks: the largest peak contains those dimers for which the *N*_HB–dim0_ fluctuates around 4, corresponding to the full starting number of HB–dim (the self-complementary dimer assembly shape is shown in Fig. 2c); the second peak indicates that a sample of dimers breaks, forming structural defects. Based on previous results on BTA supramolecular polymer dynamics,^44^ we then differentiate how many *N*_HB–dim0_ are preserved between tip and backbone dimers to identify which ones contribute to the formation of defects. Thanks to this classification, we notice that the number of HB–dim0 in the backbone is overall very stable, oscillating within the main peak of the KDE distribution (*N*_HB–dim0_ ∼ 4 in Fig. 2e), thereby demonstrating that dimer rupture events do not occur in the backbone. In contrast, the *N*_HB–dim0_ computed for specific tip dimers fluctuates from 4 to 0, revealing that full dimer breakage takes place here. In the cases of pre-assembled stacks formed by 20, 10, and 5 dimers, the KDE distribution of the tips (Fig. 3a) is also featured by two main peaks, indicating that intermediate states with *N*_HB–dim0_ from 1 to 3 are more ephemeral. In contrast, the 2D stack, where all four monomers are formally part of the tips, displays a constantly fluctuating *N*_HB–dim0_, alternating defect generation and self-healing, with intermediate dimerization states. Therefore, the 2D stack, where distinction between the backbone and tips is absent, shows a substantially higher monomer exchange.

The unique behavior of HB–dim depending on whether the dimers are arranged as tip or backbone suggests a crucial role of the physical environment in which the defects occur, specifically the key contribution from the competitive solute–solvent interaction. This is validated by a clear correlation between the time-averaged *N*_HB–dim0_ and the solvent-accessible surface area (sasa) associated with each monomer: the monomers that exhibit a larger contact with water (higher sasa) are more prone to dimer disassembly (lower 〈HB–dim〉) and hence to defect formation (Fig. 3b). The results achieved at this stage definitely confirm that the physical-chemistry source behind defect generation in the UPy-C6-u supramolecular polymers is the hydration; nevertheless, the limited time and space scales considered in these standard MD simulations make it difficult to generalize these findings. For this reason, we used the infrequent WT-MetaD technique to stimulate the rupture of a dimer bond in a 20D stack. This allows estimating the characteristic time associated with defect generation from either backbone or tip dimers. This analysis shows that the formation of a defect from a tip dimer (Fig. 3c, blue curve) is 7 orders of magnitude faster than the formation of a defect from a backbone dimer (Fig. 3c, red curve). Such a difference suggests that tip dynamics also dominates real systems where the tip-to-backbone ratio is lower than in our model supramolecular polymers.

Although the investigation around the number of HB–dim0 allows the detection of initial dimer disassembly and defect formation, in general, such an analysis is not suitable to capture the self-healing events, as *N*_HB–dim0_ cannot detect the dimerization of monomers not coupled in the starting supramolecular polymer (see also discussion in the Methods section). This is, for example, the case reported in Fig. 3d where we compare some configurations of the 10D stack along 1 μs of MD. After 100 ns, one of the tip dimers (in dark gray in Fig. 3d) disassembles, and one of the two unbound monomers slides along the supramolecular polymer, stacking on the opposite end. On approaching 1 μs, this monomer self-assembles with one of the monomers at the tip (in yellow), forming 4 complementary hydrogen bonds (number of HB–dim = 4). This is an example of defect self-healing, which characterizes the properties of this system. A similar dynamics also occurs in the 20D stack (Fig. S2 in SI). To systematically study the mechanisms of defect formation and self-healing, we analyze the dynamics of the system from a different viewpoint. Recently developed descriptors, coupled with ML tools, have been shown to accurately capture diverse structural environments within a self-assembly, including the probability of building blocks to transfer among the detected domains.^52,65^ Dynamic environments, on the other hand, have been directly extracted by using descriptors pointing out the time evolution of neighborhood environments.^53,61,62,66^ In the latter context, time smooth overlap of atomic position (τSOAP)^61^ focuses on the variations in the supramolecular structure of the system along the MD trajectory. More in detail τSOAP provides a scalar quantity (normalized from 0 to 1) that identifies the rate of structural rearrangements that occurred in the surroundings of selected centers. These centers are identified with specific groups, defining the main interactions between building blocks, thus providing a useful classification of the monomer exchange dynamics in supramolecular structures.^61^ In our case, we locate the centers of τSOAP calculation at the center of mass of the 4 atoms forming the HB–dim so that, as shown in our previous results, the arrangement of different centers is informative not only about the formation of dimers but also about the stacking. With this definition, τSOAP captures the dynamic arrangement of the UPy-C6-u interacting groups (see the Methods section for further details). Analyzing the statistics resulting from the time evolution of τSOAP for each monomer, we can classify the main dynamic domains featured in the supramolecular polymer.

**Fig. 4:**
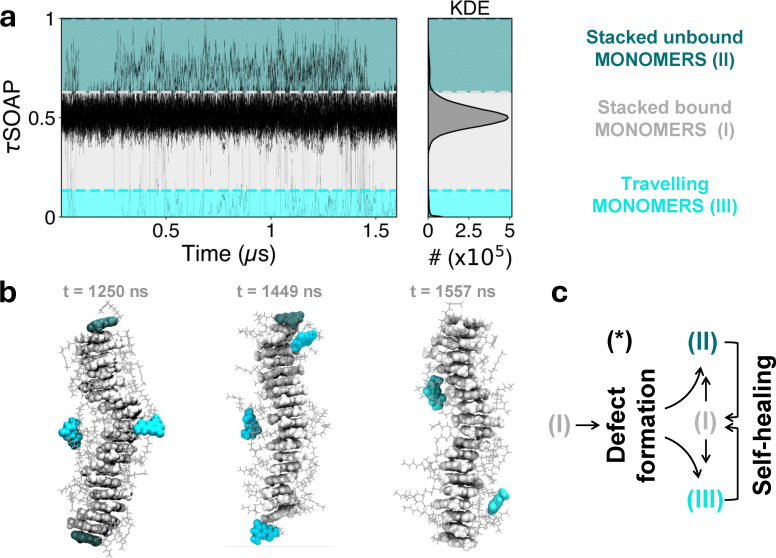
Monomer exchange pathway. (a) *τ* SOAP time series computed on the interacting centers of each monomer of the 20D UPy-C6-u supramolecular polymer in Fig. 2a, along a 1.6 μs MD. The KDE distribution of the data is shown on the right. The cluster analysis carried out on the τSOAP data detects three domains: (I) stacked bound monomers (light gray), (II) stacked, unbound monomers (teal), and (III) travelling monomers (cyan). (b) Three emblematic MD snapshots of the 20D stack, where monomers are colored according to the domains identified. (c) Scheme of possible monomer exchange pathways: starting from an ideal configuration of stacked dimers into a supramolecular polymer (I), a defect formation induces either stacked unbound (II) or travelling monomers (III), which eventually may exchange their configurations or self-heal by restoring a new stacked dimer (I).

**Fig. 5:**
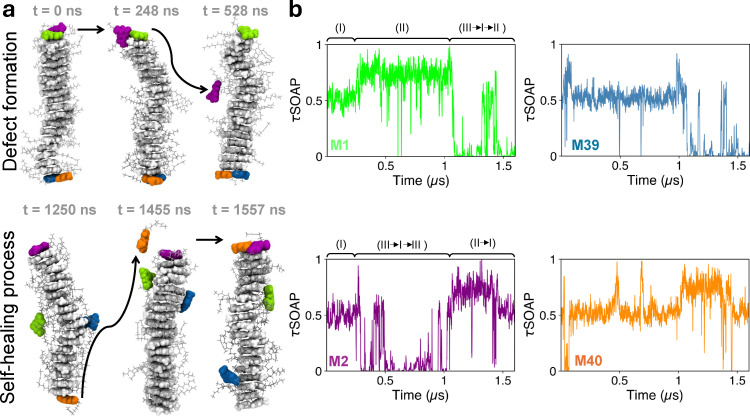
Monomer exchange pathway. (a) MD snapshots of the 20D stack. The backbone is colored in light gray, while the initial tip monomers are evidenced by different colors: M1 in green, M2 in purple, M39 in blue, and M40 in orange. The disassembly of the M1–M2 dimer is shown on the top side, showing defect formation. At 248 ns, the initial dimerization HBs break apart; then M2 starts sliding on the supramolecular polymer, while M1 remains unbound. The self-healing process instead is clarified following the M40 pathway on the downside: the decisive disassembly of the M39–M40 dimer occurs at around 1 μs, then M39 starts travelling, while M40 remains stacked and unbound. At 1455 ns M40 detaches from the supramolecular polymer and reaches M2 monomer, forming a new dimer. (b) τSOAP time series for M1, M2, M39 and M40 as defined in (a). Here, several pathways of defect creation and self-healing can be identified, clarifying the mechanisms of monomer exchange dynamics.

We thus simulate the 20D stack system for 1.6 μs, computing the τSOAP descriptor for each of the 40 monomers along the MD trajectory. The time evolution of τSOAP, together with its KDE, shows a data region particularly dense around τSOAP = 0.5, identifying the most probable dynamic state, while sparse fluctuations arise both below and above this dense data distribution (Fig. 4a). A proper cluster analysis of these data series detects the presence of three domains, one corresponding to the more dense data region and the other two above and below, respectively. Such cluster analysis also allows us to associate the identified dynamic domains to monomers that are found in a different structural state: (I) the stacked bound monomers, which form the most populated cluster characterized by an intermediate value of τSOAP, *i.e.*, a moderate rate of structural rearrangement of the environment (light gray cluster in Fig. 4a); (II) the stacked unbound monomers, which manifest the highest dynamics in terms of neighborhood reconfiguration (teal area in Fig. 4a); and (III) the monomers dissolved in solution, or sliding along the supramolecular polymer surface, which correspond to the lowest values of τSOAP (cyan area in Fig. 4a). In configuration III, the interacting centers are far from the others, thus, their environment appears static from our definition of τSOAP (cyan region in Fig. 4a). Three MD snapshots of the 20D stack at distinct time steps are reported with the monomers' colors corresponding to the classification in Fig. 4a. In this graphical representation, the ideal assembly configuration (I), characterizing the bound monomers, as well as the defect events, identified as either (II) or (III) states can be clearly visualized. Based on this classification, we can now interpret the τSOAP signal relative to each monomer (center): the defect formation events take place when a monomer transfer occurs from I to II or from I to III—and self-healing events arise when a monomer moves from II or III to domain I (Fig. 4c). Fig. 5 shows some emblematic examples of monomer exchange dynamics. For instance, M1 and M2 are two tip monomers initially bound, as distinctly confirmed by their τSOAP values, averaging around 0.5 for the first 250 ns (green and purple τSOAP signals in Fig. 5b and relative MD snapshots in Fig. 5a). Then, after 250 ns, a defect forms and while M1 remains bound to the tip (with its τSOAP value transitioning to higher values, II), M2 starts sliding along the supramolecular polymer (with τSOAP value shifting towards 0, III). After 1 μs, we observe a self-healing event, in which M2, which in the meanwhile has reconfigured as a stacked unbound monomer (domain II), dimerizes with M40, which travels toward M2 from the other end of the supramolecular polymer (the orange profile in Fig. 5b and final MD snapshot in Fig. 4a).

Overall, this second analysis also confirms that monomer exchange dynamics concentrates at the tips of the supramolecular polymers, highlighting that both defect creation and self-healing preferentially occur at the ends (Fig. S2 in the SI). Coherent results are also obtained by combining τSOAP and LENS descriptors (Fig. S3 in the SI).^53,62^ This observation is consistent with the relatively slow dynamics of UPy-C6-u supramolecular polymers detected in the experiment.

**Fig. 6:**
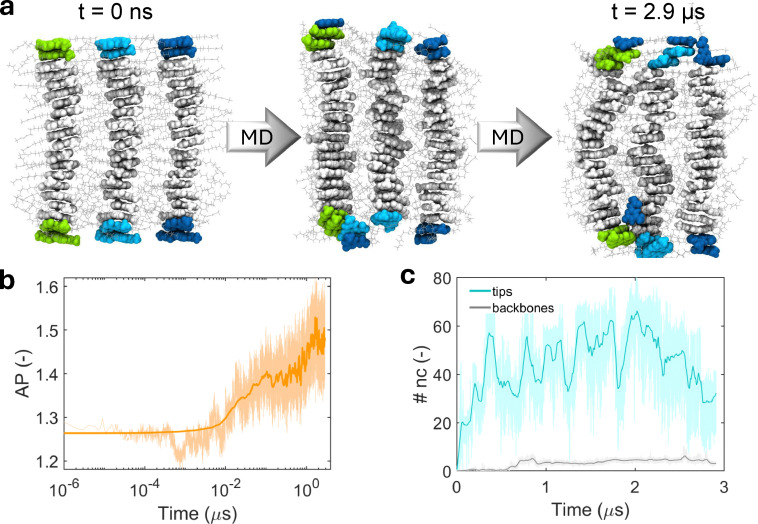
Inter-supramolecular polymer monomer exchange dynamics. (a) Three snapshots of AA-MD simulating three parallel 20D stack placed next to each other. Monomers initially located at the tips of each supramolecular polymer, as well as the first neighbors, are colored in green, cyan and blue, depending on the supramolecular polymer they belong to. The other, backbone monomers are colored in gray. The inter-supramolecular polymer dynamics is localized at the tips. (b) Aggregation propensity (AP, see text for the definition) of the three stack along the AA-MD simulation. (c) Average number of inter-supramolecular polymer contacts per monomer (nc) in case of tips/subtips (cyan curve) and backbones (gray curve).

We finally explored how the monomer exchange dynamics is affected when multiple UPy-C6-u stacks (Fig. 6) interact to form fibers, typical of the UPy supramolecular structure.^35,43,58,63^ We therefore carried out an unbiased MD simulation of three 20D pre-assembled stacks initially placed next to each other, following their interaction along the trajectory. As shown by the sequential snapshots reported in Fig. 6a, few events of inter-supramolecular polymer exchange were captured within a 3 μs timeframe.

Entering more in depth into the inter-supramolecular polymer dynamics we observe that the aggregation propensity (AP), namely the ratio between the sasa of three ideally isolated stacks over the sasa of the assembly computed at time *t*, signals the tendency of the supramolecular polymers to aggregate in fibers, progressively reducing their solvent exposure (Fig. 6b). This aggregation is mainly driven by side-chain interactions, as shown by the relatively small number of inter-supramolecular polymer contacts formed by the core UPy motifs, *i.e.* excluding side-chain contacts (Fig. 6c). Interestingly, monomer exchange within the fiber occurred almost exclusively at the supramolecular polymer tips. Separating the average number of inter-stack contacts “nc” established by each tip monomer (end dimers and their first neighbors) from those established by backbone monomers revealed a striking difference between the two. The value of tips’ nc mostly fluctuates within the 40–60 range, whereas the backbone's nc mostly remains around 5 (Fig. 6c). Besides this quantitative evidence, MD snapshots visually confirm that exchanges are localized at the tips (Fig. 6a). Moreover, by performing long MD simulations (7 μs) of three infinite supramolecular polymers at *T* = 343 Kelvin, we could observe that backbone exchanges are not strictly forbidden, but they are far less frequent than tip exchanges (Fig. S4). This evidence supports our interpretation of why UPy supramolecular polymers display slower dynamics compared to other supramolecular polymers such as BTA.^35,41^ Our analysis also indicates that the monomer sliding observed along the surface of an isolated chain becomes less favorable in fibers, where side-chain interactions between adjacent UPy stacks hinder such motion. These simulations on multiple supramolecular polymers therefore provide valuable context for interpreting single-chain monomer exchange results in the framework of hierarchical structures formed by UPy motifs.

## 2. Conclusions

We here report for the first time a computational MD study on the monomer exchange dynamics of UPy-C6-u supramolecular polymers in aqueous solution. In particular, we shed light on the processes of defect creation and self-healing pathways, which are key aspects for monomer motion in supramolecular polymers.

First, the dimerization free energy surface between two UPy-C6-u monomers in water has been investigated to assess the supramolecular architecture of the system, based on quadruple hydrogen bonds, *i.e.* HB–dim, creating dimer units that stack into supramolecular polymers. Structural analysis carried out on such dimers' stacks of distinct sizes has highlighted a cooperative effect, by which the quadruple HB–dim appears to be more stable in longer supramolecular polymers, with subsequent increased stability of the entire structure.

The monomer exchange mechanism in UPy supramolecular polymers was then explored more in detail. By following the initial number of HB–dim0 along MD trajectories, we found that the dimers' disassembly seen as a consistent variation of the number of HB–dim0 involves exclusively tip monomers rather than backbone. To explain this evidence, the solvent accessible surface area was calculated for each monomer along the MD trajectory. The resulting data demonstrate a correlation between hydrogen bond breaking and monomer hydration, thereby suggesting that competitive solute–solvent interactions are the main driving force for dimer rupture, *i.e.* defect formation. This result is also quantitatively supported *via* infrequent WT-MetaD simulations, showing that dimer defect formation occurs several order of magnitude faster at the tips than in the backbone.

We then employed τSOAP,^61^ a recently developed descriptor of atomic environment dynamics, to obtain further insights into the most probable defect and self-healing mechanisms taking place along these supramolecular polymers. Specifically, τSOAP coupled with ML tools allow classifying the different monomers according to the dynamics of their supramolecular surroundings, thereby unveiling possible pathways of monomer exchange events. We finally presented AA-MD simulations of UPy supramolecular fibers, showing indications on how monomer exchange dynamics takes place when multiple UPy stacks aggregate. Overall, our results show that the origin of UPy supramolecular polymers dynamics always relies on the mechanism of dimers' disassembly, which, while leading to either traveling or stacked monomers (defect creation), offers a suitable local environment for self-healing (defect resolution). In conclusion, beyond the specific case study, our combined computational approaches establish a modeling strategy capable of systematically investigating hierarchical self-assembly in supramolecular systems. Although experimental techniques have made it possible to quantify the supramolecular dynamics (*e.g.* estimating a 10% of monomer exchange per hour in UPy supramolecular polymers *versus* 30–40% per hour in BTA supramolecular polymers^35,41^), in this study, we complement the experiments by unveiling the most favorable local environment for dimer breakage, the mechanism of monomer exchange, and the underlying principle of self-healing.

## 3. Methods

### 3.1. All-atom molecular dynamics (AA-MD) simulations

The atomistic models of UPy monomers, including both UPy without functionalization and UPy-C6-u, were built with Avogadro^67^ following the chemical structure of the molecules. Gaussian^68^ tool, based on the HF/6-31G*, was used to estimate the generated electrostatic potential, and then the RESP^69^ method was applied to obtain the partial charge distribution within the molecule. The complete parameterization was based on the General AMBER Force Field (GAFF),^70^ using Antechamber.^71^

#### 3.1.1. Self-assembly of UPy monomers

The self-assembly MD simulation of 42 non-fuctionalized UPy monomers (*i.e.*, without side-chain R1) was carried out in GROMACS 2021.^72^ First, the parameterized UPy monomers were randomly dispersed in a 10 × 10 × 10 nm^3^ box filled with water molecules described using the TIP3P model^73^ and periodic boundary conditions were applied in all box directions. The non-bonded interactions among monomers, including van der Waals and short-range electrostatic interactions, were evaluated within a cut-off radius of 1.4 nm, while for the remaining long-range interactions, a particle-mesh Ewald summation was applied to resolve electrostatics in the Fourier space. Two equilibration steps were performed to reach the thermodynamic conditions of 298 K and 1 bar. The self-assembly simulation, lasting 1 μs, was performed by using the v-rescale thermostat^74^ (*τ*_T_ = 0.1 ps) coupled with the c-rescale barostat^75^ (*τ*_p_ = 0.1 ps).

#### 3.1.2. Pre-assembled UPy-C6-u supramolecular polymers

We promoted the dimerization of two UPy-C6-u monomers and their self-assembly in the axial direction, forming UPy-C6-u stacks of distinct size from 2 to 20 dimers. We studied the stability of a single UPy-C6-u supramolecular polymer in aqueous solution *via* classical AA-MD simulations carried out with the open-source software GROMACS 2021.^72^ Each single pre-assembled stack was first solvated in a 5 × 5 × 5 nm^3^ box filled with water molecules described using the TIP3P model^73^ and periodic boundary conditions were applied in all box directions. Note that for the 20D stack, a 10 × 10 × 10 nm^3^ box was considered. The non-bonded interactions among monomers, including van der Waals and short-range electrostatic interactions, were evaluated within a cut-off radius of 1.4 nm, while for the remaining long-range interactions, a particle-mesh Ewald summation was applied to resolve electrostatics in the Fourier space. Our MD protocol consisted of a first step of energy minimization and two consequent equilibration steps. Initially, to reach an equilibrium temperature of 298 K, we applied the canonical ensemble (NVT) for 2 ns using a Maxwell Boltzmann speed distribution and the v-rescale thermostat^74^ with *τ* = 0.1 ps. Subsequently, we set the isothermal–isobaric (NPT) ensemble for 2 ns at an equilibrium pressure of 1 bar and an equilibrium temperature of 298 K. In this step, we used the previous thermostat coupled with the c-rescale barostat^75^ with a time constant of 2 ps. During the equilibration steps, the UPy-C6-u atoms were restrained in their initial positions using a harmonic potential with a force constant of 1000 kJ mol^−1^ nm^−2^. Once the desired thermodynamic conditions were reached, the restraint was removed, and a 400 ns-long MD run (integration step d*t* = 0.002 ps) was carried out by maintaining the temperature at 298 K with a Noose–Hoover thermostat (*τ*_T_ = 0.8 ps) and a pressure at 1 bar (*τ*_p_ = 2 ps) by imposing the Parrinello–Rahman barostat^76^ Along the MD simulation, the LINCS algorithm was employed to restrain the covalent bonds involving hydrogen atoms.

To compare the stability of the four supramolecular polymers, we first analyzed the production run trajectories by computing the radial distribution function between UPy-C6-u monomers. The HB–dim were estimated in PLUMED 2.6.^77,78^ taking into account the coordination (*R*_0_ = 0.12 *D*_0_ = 0.27) among the colored atoms in the zoomed-in view of Fig. 2c. Note that, while performing this estimation, we kept the initial dimer configuration through the complete trajectory analysis. The τSOAP descriptor was instead applied to each monomer of the 20D stack, and specifically on the center of mass of the oxygen and nitrogen atoms involved in possible HB–dim (zoom in Fig. 2c). Thus, for each individual center *i*, τSOAP_*i*_ (*t*) monitors the *i*-th local environment changes in terms of neighbor monomers' arrangement along the trajectory, ranging from 0 to 1 for static to highly dynamic neighborhoods, respectively. The instantaneous τSOAP value is defined as:1
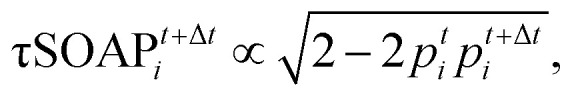
where *p*^*t*^_*i*_ is the full SOAP feature vector associated with the *i*-th individual center within a certain cutoff neighborhood (*r*_cut_) at the time step *t*, as described in detail in ref. 61. Here, *r*_cut_ = 0.6 nm was employed. In brief, τSOAP_*i*_ (*t*) tracks the variations of the *i*-th SOAP vector over time, that is, to what extent the molecular environment related to each center changes at every consecutive time interval Δ*t* in terms of SOAP power spectrum. The unsupervised clustering algorithm of Gaussian Mixture Models^79^ was finally adopted to rationalize the data and to identify the dominant molecular environments in the supramolecular polymer. Similar outcomes are also achieved by applying the recent LEAP analysis,^62^ combining LENS^53^ and τSOAP descriptors (Fig. S3 in the SI).

### 3.2. UPy-C6-u dimerization free energy surface

To explore dimerization FES, which shows the thermodynamic phase space of two UPy-C6-u monomers interacting in aqueous solution, we performed extensive 355 ns-long well-tempered MetaDynamics (WT-MetaD) simulations.^64^ We selected as collective variables (CVs) (i) the number of HB–dim (*R*_0_ = 0.12 *D*_0_ = 0.27), and (ii) the core–core distance (see Fig. 1d). The latter CV represents the distance between the centers of the two UPy-C6-u cores. Note that the coordination number was computed as implemented in PLUMED 2.6.^77,78^ We chose 10 as a bias factor with an initial Gaussian height of 1.5 kJ mol^−1^, and a width of 0.5 nm for both the distance and the coordination number, respectively. The Gaussian deposition rate was set to 5000 MD step^−1^, *i.e.*, every 10 ps. After reaching convergence, we reweighted the FES using the Tiwary–Parrinello estimator^80^ on the same CVs. The WT-MetaD simulations were performed using GROMACS 2021^72^ and PLUMED 2.6.^77,78^

### 3.3. Infrequent WT-MetaD simulations

The formation of defects along the supramolecular polymer is a rare event in the timescales effectively accessible using atomistic models. As demonstrated, the real (unbiased) dynamics of an event is related to the transition time associated with events activated by infrequent WT-MetaD simulations (biased dynamics).^60,80,81^ This approach is particularly convenient as it allows one to directly extract information on the kinetics of the activated transition from the biased WT-MetaD simulations. Adapting this approach, we calculated the characteristic timescales, *τ*, for defect formation both within the backbone and on the tips of the 20D stack. In particular, we run multiple infrequent WT-MetaD simulations where the systems undergo a transition from HB–dim = 4 to HB–dim = 0. The unbiased transition time (*t*) of each transition can be calculated from each WT-MetaD run as:

2 *t* = *t*_WT-MetaD_〈e^*βV*(*s*(***R***,*t*))^〉_WT-MetaD_

where, *V*(*s*(***R***), *t*) is the time-dependent bias, the exponential brackets is averaged over the WT-MetaD run, and *β* is *kT*^−1^. The characteristic time scale, *τ*, of defect formation is then calculated by fitting the cumulative distribution function (CDF) with a Poisson-like cumulative probability:3
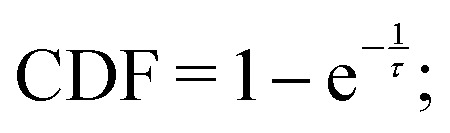


Fig. 2d shows the CDF profiles of the defect formation at the supramolecular polymer tip (blue curve), and backbone (red curve).

## Author contributions

C. P. and A. C. designed the setup and the computational framework. L. R. and P. D. provided the case study. A. C. and C. C. carried out the simulations and the postprocessing of the data. All the authors worked on the interpretation of the results. C. P. supervised the project. All the authors wrote and approved the final manuscript.

## Conflicts of interest

There are no conflicts to declare.

## Data Availability

The data supporting this article have been included as part of the supplementary information (SI). The SI contains the planar arrangement of the dimers forming 20D and 2D supramolecular polymers, the LENS and τSOAP time series corresponding to the interacting centers of each monomers forming the 20D UPy-C6-u supramolecular polymer, and the AA-MD snapshots of three bundled supramolecular polymers at 343 K. See DOI: https://doi.org/10.1039/d5tb01272d. All data and materials related to the simulation MD trajectories and the analysis conducted herein are available at: https://doi.org/10.5281/zenodo.17236935.
